# Newborn Screening for Mucopolysaccharidosis Type II in Illinois: An Update

**DOI:** 10.3390/ijns6030073

**Published:** 2020-09-03

**Authors:** Barbara K. Burton, Rachel Hickey, Lauren Hitchins

**Affiliations:** 1 Department of Pediatrics, Ann & Robert H. Lurie Children’s Hospital of Chicago, Chicago, IL 60611, USA; rahickey@luriechildrens.org (R.H.); lhitchins@luriechildrens.org (L.H.); 2 Department of Pediatrics, Northwestern University Feinberg School of Medicine, Chicago, IL 60611, USA

**Keywords:** mucopolysaccharidosis type II, pseudodeficiency, newborn screening

## Abstract

Mucopolysaccharidosis type II (MPS II, Hunter syndrome) is a rare, progressive multisystemic lysosomal storage disorder with significant morbidity and premature mortality. Infants with MPS II develop signs and symptoms of the disorder in the early years of life, yet diagnostic delays are very common. Enzyme replacement therapy is an effective treatment option. It has been shown to prolong survival and improve or stabilize many somatic manifestations of the disorder. Our initial experience with newborn screening in 162,000 infants was previously reported. Here, we update that experience with the findings in 339,269 infants. Measurement of iduronate-2-sulfatase (I2S) activity was performed on dried blood spot samples submitted for other newborn screening disorders. A positive screen was defined as I2S activity less than or equal to 10% of the daily median. In this series, 28 infants had a positive screening test result, and four other infants had a borderline result. Three positive diagnoses of MPS II were established, and 25 were diagnosed as having I2S pseudodeficiency. The natural history and the clinical features of MPS II make it an ideal target for newborn screening. Newborn screening was effective in identifying affected infants in our population with an acceptable rate of false positive results.

## 1. Introduction

Mucopolysaccharidosis type II (MPS II, Hunter syndrome) is one of the most common of the MPS disorders with an estimated incidence of 1 in 100,000 to 1 to 170,000 births [1,2]. It is a progressive multisystemic lysosomal storage disorder which results in significant morbidity and premature mortality. MPS II is the result of a deficiency of the enzyme iduronate-2-sulfatase (I2S), which leads to progressive accumulation of the glycosaminoglycans (GAGs) dermatan sulfate and heparan sulfate. Infants born with this condition are typically normal at birth but begin to develop signs and symptoms of the disorder in the early years of life. Despite this, diagnostic delays are very common due to the rarity of the disorder and the fact that many of the early signs and symptoms are ones that are common in the general pediatric population [3]. There is a spectrum of severity of MPS II with about two-thirds of the patients having the severe form of the disorder associated with cognitive impairment and progressive cognitive decline. These patients typically succumb to the condition in their early 20s. Patients with the attenuated form of the disorder have no cognitive impairment and may survive into middle age or beyond but can have significant disabilities related to their somatic disease.

Enzyme replacement therapy (idursulfase) has been available for the treatment of MPS II for over a decade in the United States and many other countries around the world. It is effective in prolonging survival [4] and improving or stabilizing many of the somatic manifestations of the disorder [5]. Hematopoietic stem cell transplantation has also been performed in a number of cases [6,7] and may provide some cognitive benefit if performed very early in life, although experience is limited, and it is clearly not as efficacious for the central nervous system disease as it is in MPS I. Experience with other lysosomal storage disorders has revealed that earlier treatment, including treatment prior to the onset of clinical symptoms, is typically associated with improved outcomes [8,9]. This is not surprising, since progressive disease may lead to irreversible organ damage or dysfunction, which could be prevented with early treatment. While there is limited experience described in the literature in the presymptomatic treatment of MPS II, early treatment has been shown to be safe [10] and, based on the progressive nature of the disease, is likely to be beneficial.

Newborn screening for MPS II is currently ongoing in Taiwan [11,12] and in a number of pilot projects in Japan. Illinois became the first state in the United States to implement population-based newborn screening for MPS II in December 2017. The initial experience with 162,000 infants has been previously reported [13]. In this communication, we update that experience by reporting the findings in 339,269 infants screened between 11 December 2017 and 29 February 2020.

## 2. Materials and Methods

Measurement of I2S activity was performed as previously described [13] in the Newborn Screening Laboratory of the Illinois Department of Public Health using the same dried blood spot samples submitted for the other newborn screening disorders. A positive screen was defined as I2S activity less than or equal to 10% of the daily median. Immediate referral to a designated consultant for diagnostic testing was recommended for infants with a positive result. Samples with I2S activity greater than 10% but less than or equal to 13% of the daily median were classified as borderline, and a second filter paper specimen was requested. If the result on the second sample was again borderline or positive, the infant was referred for diagnostic testing. Diagnostic testing was performed at commercial laboratories at the discretion of the consultant evaluating the patient. Molecular analysis of the IDS gene was performed by Sanger sequencing at either the Greenwood Genetic Center or the Mayo Medical Laboratory. Data on the follow-up testing performed on screen positive infants were provided to the state Newborn Screening Follow-up Program and were provided in a de-identified format to the authors. For the patients evaluated by the authors, complete data were available, but this was not uniformly the case for patients evaluated at other centers.

## 3. Results

A total of 339,269 infants were screened between 17 December 2011 and 29 February 2020. Twenty-eight infants had a positive screening test result, and four other infants had a borderline result. All 32 infants (0.009% of the total screened) were male. In one case of a borderline result, the I2S activity on the second sample was normal, thus no further testing or evaluation was conducted. In the other three cases, the second sample also yielded a borderline or positive result, thus the infants were referred for diagnostic testing. In total, therefore, 31 infants were seen for diagnostic evaluation. Three positive diagnoses of MPS II were established. Of the remaining 28 infants, three had normal I2S activity in plasma on follow-up testing. The remaining 25 all had low plasma I2S activity and, after subsequent evaluation and additional laboratory testing, were diagnosed as having I2S pseudodeficiency.

The three infants with confirmed diagnoses of MPS II all had I2S activity on the newborn screening sample of 0–2% of the daily median. All three had elevated total urine GAGs and significant elevations of both dermatan sulfate and heparan sulfate. All had previously reported pathogenic mutations in the *IDS* gene: c.1403G>A, p.R468Q; c.257C>T, p.P86L; and c.1506G>A, p.W502X. One of the infants had a two-year-old male sibling who was described as having developmental delay and on physical examination had typical features of MPS II. The diagnosis was confirmed biochemically in the older sibling as well. Therapeutic options, including hematopoietic stem cell transplantation and enzyme replacement therapy, were discussed with all three families. Two of the affected infants were started on idursulfase therapy at four and six weeks of age. The third family has thus far declined treatment.

The patients with presumed pseudodeficiency are listed in Table 1. Enzyme activity on the newborn blood spot ranged from 1–13% of the daily median in these patients, and seven of the 25 had a level below 5% of the daily median. Two of them had initial values of 1–2% of the daily median consistent with what was observed in the three affected infants. Several infants had two separate filter paper samples submitted for analysis, in which case both results are listed. All 25 infants had normal urine or dried blood spot GAGs except case #12, who had mildly elevated urine GAGs of 62.2 mg/mmol creatinine (normal: <53 mg/mmol creatinine), although it was noted that it was a dilute specimen. Several other infants had minor elevations of dermatan sulfate and/or heparan sulfate despite normal total GAGs. If it is indicated in Table 1 that urine GAGs were not done, the patient had dried blood spot GAGs done by a reference laboratory, which were reported only as normal. When the urine GAG results are listed as normal, this is the only information that was provided by the consultant evaluating the patient. In all 25 infants, sequencing of the *IDS* gene was performed, and in 24 of these, a variant was detected. Ten of these had pseudodeficiency variants previously reported from Taiwan [11,12] (c.1499C>T, p.T500I; c.1478G>A, p.R493H; c.890G>A, p.R297H and the linked variants c.684A>G, p.P228P and c.851C>T, p.P284L); eight of these infants were of Asian descent. Two other Caucasian infants had the same variant c.1055A>G, p.D352G, and both had very low dried blood spot I2S activity (2% and 3% of daily median) as well as plasma I2S activity within the affected range. Both had normal urine GAGs. Two others, both African-American, had the variant c.754G>T, p.D252Y. Both of these infants had dried blood spots (DBS) I2S activity just below the cutoff and plasma I2S activity well above the affected range. All of the other infants had unique variants not previously described and were representative of all racial and ethnic groups. Family studies were conducted in many of the cases, and three of the infants with presumed pseudodeficiency (Cases 3, 12, and 13) had clinically unaffected maternal grandfathers with the same variant present in the infant as well as similar biochemical findings. Based on the criteria established by the American College of Medical Genetics and Genomics (ACMG), all of the variants listed in Table 1 would be classified as variants of unknown significance.

In addition to the three affected infants and the 25 infants with presumed pseudodeficiency for I2S, molecular testing was also performed on two screen positive infants who had normal plasma I2S activity. One of these had the c.1499C>T, p.T500I pseudodeficiency variant, and the other had a novel variant- c.364T>G p.Y122D.

## 4. Discussion

The natural history and the clinical features of MPS II make it an ideal target for newborn screening efforts. Affected infants typically appear normal at birth but develop multiple signs and symptoms of this multisystemic disorder in early childhood. Treatment is available, but diagnosis is often delayed for many years, leading to lengthy delays in the initiation of therapy and the development of potentially irreversible disease. Newborn screening prevents diagnostic odyssey and delayed diagnosis experienced by many families who have a child with this disorder and will likely lead to improved outcomes. Current treatment options include enzyme replacement therapy and hematopoietic stem cell transplantation, but numerous other treatments targeting both somatic disease and the central nervous system disease are in clinical trials or in pre-clinical development. Although the benefits of presymptomatic enzyme replacement therapy or transplantation have thus far been documented only in a small number of affected infants diagnosed prior to clinical recognition [14], experience with other lysosomal disorders would suggest that this will likely be beneficial, since many of the findings of the disorder are likely to be more amenable to prevention than to reversal. Even in the absence of such evidence of early efficacy in preventing clinical symptoms, early diagnosis is likely to improve outcomes through surveillance for disease complications and recognition of the anesthetic risks associated with the disorder. Patients with MPS II typically undergo multiple surgical procedures in the early years of life, often prior to recognition of the diagnosis [15]. Since airway narrowing and post-operative complications are common, it is essential that such surgery be performed in a facility equipped to manage a difficult airway.

In the current series, 29 of 30 screen positive infants who had molecular testing had a variant detected in the *IDS* gene. Three of these were known pathogenic variants, and these three infants were all clearly affected based on both low plasma enzyme activity and elevated urine or dried blood spot GAGs. The remaining infants were all presumed to have I2S pseudodeficiency, since no evidence of substrate accumulation was demonstrated. Many but not all of them had plasma I2S enzyme activity that was below normal but higher than that observed in affected infants. There remains some uncertainty with regard to whether it can be positively concluded that all of these infants are unaffected. One infant in our series had the c.1601A>G variant, which has not been previously reported. However, an MPS II patient in Japan with an attenuated phenotype has been reported with a different missense change at the same nucleotide position (c.1601A>T) [16]. It seems very unlikely that our patient is affected in light of his plasma I2S activity, which was close to the normal range at 123.6 nmol/4hr/mL (normal: >155). Similarly, while the linked variants c.684A>G and c.851C>T have been reported from Taiwan to result in pseudodeficiency, an attenuated patient has been reported from Japan with the c.851C>T variant alone. Personal communication with the authors of that report revealed that further assessment of the patient led them to conclude that he was unaffected with MPS II. In order to feel confident that normal urine or dried blood spot GAGs eliminate the possibility of a diagnosis of MPS II, more data on urine and dried blood spot GAGs in newborn attenuated patients are needed. Unfortunately, these samples are very difficult to obtain.

The incidence of pseudodeficiency for I2S cannot be accurately determined from our data alone, but it is clearly more common than true deficiency. It also appears to occur in all racial and ethnic groups, although some specific pseudodeficiency alleles appear to be common in the Asian populations. This is in contrast to iduronidase pseudodeficiency in North America, which occurs primarily in the African-American population. If it can be demonstrated that dried blood spot GAGs are clearly elevated in all MPS II patients at birth, even those with attenuated phenotypes, then this may provide a valuable second tier test for use in newborn screening to eliminate the need for further evaluation of these pseudodeficiency patients. Even without second tier testing, however, newborn screening has been very effective in identifying affected infants in our population with an acceptable rate of false positive results.

## Figures and Tables

**Table 1:** Results of follow-up testing in infants with iduronate-2-sulfatase (I2S) pseudodeficiency.

Case Number	DBS I2S (% Daily Median)	Diagnostic I2 (Reference Range)	Urine GAGs ^3^ (Normal 0–53)	IDS Sequence Variant
1	10	31.1 ^1^ (≥155)	24.81	c.863T>C, p.I288T
2	9	50.9 ^1^ (≥155)	28.92	None
3	4	30.4 ^1^ (≥155)	41.60	c.554C>G, p.P185R
4	6	61.6 ^1^ (≥155)	18.91	c.684A>G, p.P228P and c. 851C>T, p.P284L
5	10	60.51 ^1^ (≥155)	19.50	c.778C>T, p.P260S
6	2	5.32 ^1^ (≥155)	15.47	c.1055A>G, p.D352G
7	10	137.7 ^1^ (≥155)	22.71	c.754G>T, p.D252Y
8	7	22.56 ^1^ (≥155)	22.56	c.684A>G, p.P228P; and c.851C>T, p.P284L ^5^
9	10	100.1 ^1^ (≥155)	15.32	c.1499C>T, p.T500I
10	3	<1.5 ^2^ (>1.5)	21.30	c.1499C>T, p.T500I
11	No value	<1.5 ^2^ (>1.5)	45.50	c.1055A>G, p.D352G
12	12, 11	1.5 ^2^ (>1.5)	62.20	c.1499C>T, T500I
13	3	<1.5 ^2^ (>1.5)	43.60	c.674A>G, p.Y225C
14	1	<1.5 ^2^ (>1.5)	Normal	c.785T>A, p.V262E
15	13, 13	123.6 (≥155)	Normal	c.1601A>G, p.N534S
16	7	16.7 (≥155)	17.38	c.1478G>A, p.R493H
17	8	21.8 (≥155)	23.64	c.1409C>T, p.S470L
18	10	113.7 (≥155)	21.48	c.754G>T, p.D252Y
19	11, 7	64.2 (≥155)	Normal	c.684A>G, p.P228P and c.851C>T, p.P284L ^5^
20	8	84.6 (≥155)	Normal	c.1499C>T, p.T500I
21	3	2.86 (≥155)	Not done ^4^	c.785T>A, p.V262E
22	4	23.9 (≥155)	Normal	c.1417C>T, p.P473S
23	6	<1.5 (>1.5)	Not done ^4^	c.1478G>A, p.R493H
24	7	<1.5 (>1.5)	Not done ^4^	c.890G>A, p.R297H
25	8	<1.5 (>1.5)	Not done ^4^	c.1205A>C, p.G402A

^1^ Values expressed as nmol/4 h/mL in plasma; performed at Greenwood Genetic Center. Affected range: <15 nmol/4 h/mL. ^2^ Values expressed as nmol/h/mL in dried blood spots (DBS); performed at Mayo Medical Laboratories. Affected range: <1.5 nmol/h/mL. ^3^ Values expressed as mg/mmol creatinine. ^4^ DBS glycosaminoglycans (GAGs) were done for this case and reported as “normal”. No specific values were provided. ^5^ The two mutations were linked.
